# (3,5-Dichloro­salicylaldehyde thio­semi­carbazonato-κ^3^ *S*,*N* ^1^,*O*)(*N*,*N*′-dimethyl­formamide-κ*O*)copper(II) dimethyl­formamide solvate

**DOI:** 10.1107/S1600536808008982

**Published:** 2008-04-10

**Authors:** Yuan Wang, Zheng Liu, Jiong-Yang Gao

**Affiliations:** a Key Laboratory of Non-ferrous Metal Materials and New Processing Technology, Department of Materials and Chemical Engineering, Guilin University of Technology, Ministry of Education, Guilin 541004, People’s Republic of China

## Abstract

In the title compound, [Cu(C_8_H_5_Cl_2_N_3_OS)(C_3_H_7_NO)]·C_3_H_7_NO, the Cu^II^ atom is coordinated in a slightly distorted square-planar geometry by an O, an S and an N atom from the tridentate ligand 3,5-dichloro­salicylaldehyde thio­semi­carb­azonate ligand and one O atom from dimethyl­formamide. At the same time, the Cu atom is in contact with S and Cl atoms from another two complexes [Cu⋯S and Cu⋯Cl = 2.9791 (2)  and 3.3800 (3) Å, respectively], thereby forming a [4 + 2] coordination geometry. The crystal structure exhibits N—H⋯O and N—H⋯N hydrogen bonds.

## Related literature

For studies of thio­semicarbazone complexes containing amino acids, see: Garcia-Orozco *et al*. (2002 ▶); Seena *et al.* (2007 ▶); Valdes-Martinez *et al.* (1995 ▶); Singh *et al.* (1997 ▶); Shen *et al.* (1997 ▶); Zimmer *et al.* (1991 ▶).
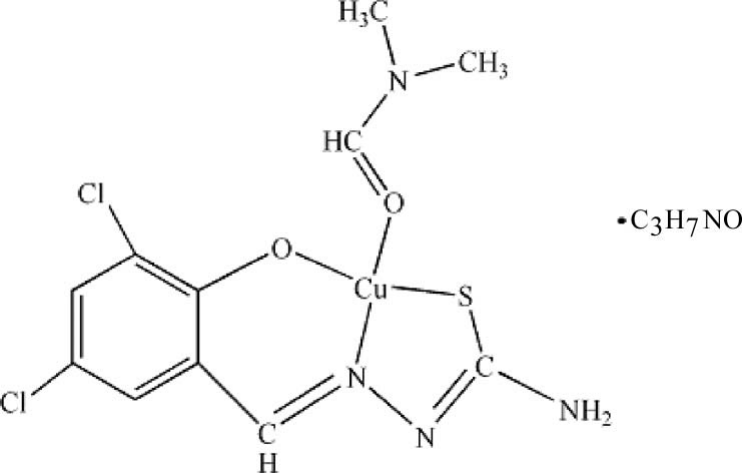


## Experimental

### 

#### Crystal data

[Cu(C_8_H_5_Cl_2_N_3_OS)(C_3_H_7_NO)]·C_3_H_7_NO
*M* *_r_* = 471.84
Monoclinic, 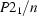

*a* = 9.4979 (10) Å
*b* = 9.8057 (12) Å
*c* = 21.744 (2) Å
β = 94.263 (2)°
*V* = 2019.5 (4) Å^3^
*Z* = 4
Mo *K*α radiation
μ = 1.47 mm^−1^
*T* = 298 (2) K
0.49 × 0.47 × 0.24 mm

#### Data collection

Bruker SMART 1000 diffractometer
Absorption correction: multi-scan (*SADABS*; Sheldrick, 1996 ▶) *T*_min_ = 0.532, *T*_max_ = 0.719
9869 measured reflections
3558 independent reflections
2587 reflections with *I* > 2σ(*I*)
*R*_int_ = 0.034

#### Refinement

*R*[*F* ^2^ > 2σ(*F* ^2^)] = 0.036
*wR*(*F* ^2^) = 0.098
*S* = 1.06
3558 reflections
235 parameters
H-atom parameters constrained
Δρ_max_ = 0.31 e Å^−3^
Δρ_min_ = −0.50 e Å^−3^

### 

Data collection: *SMART* (Bruker, 2007 ▶); cell refinement: *SAINT* (Bruker, 2007 ▶); data reduction: *SAINT*; program(s) used to solve structure: *SHELXS97* (Sheldrick, 2008 ▶); program(s) used to refine structure: *SHELXL97* (Sheldrick, 2008 ▶); molecular graphics: *SHELXTL* (Sheldrick, 2008 ▶); software used to prepare material for publication: *SHELXTL*.

## Supplementary Material

Crystal structure: contains datablocks global, I. DOI: 10.1107/S1600536808008982/om2219sup1.cif

Structure factors: contains datablocks I. DOI: 10.1107/S1600536808008982/om2219Isup2.hkl

Additional supplementary materials:  crystallographic information; 3D view; checkCIF report

## Figures and Tables

**Table:** 

Cu1—O1	1.909 (2)
Cu1—N1	1.954 (3)
Cu1—O2	1.985 (2)
Cu1—S1	2.2567 (10)

**Table:** 

O1—Cu1—N1	93.55 (11)
O1—Cu1—O2	90.55 (11)
N1—Cu1—O2	172.04 (11)
O1—Cu1—S1	176.26 (8)
N1—Cu1—S1	86.04 (8)
O2—Cu1—S1	89.43 (8)
C4—O1—Cu1	127.5 (2)
C9—O2—Cu1	123.8 (3)
C1—S1—Cu1	94.29 (11)

**Table 2:** Hydrogen-bond geometry (Å, °)

*D*—H⋯*A*	*D*—H	H⋯*A*	*D*⋯*A*	*D*—H⋯*A*
N3—H3*A*⋯N2^i^	0.86	2.14	2.992 (4)	170
N3—H3*B*⋯O3^ii^	0.86	2.08	2.886 (4)	157

Symmetry codes: (i) 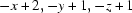; (ii) 
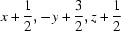
.

**Fig. 1.**
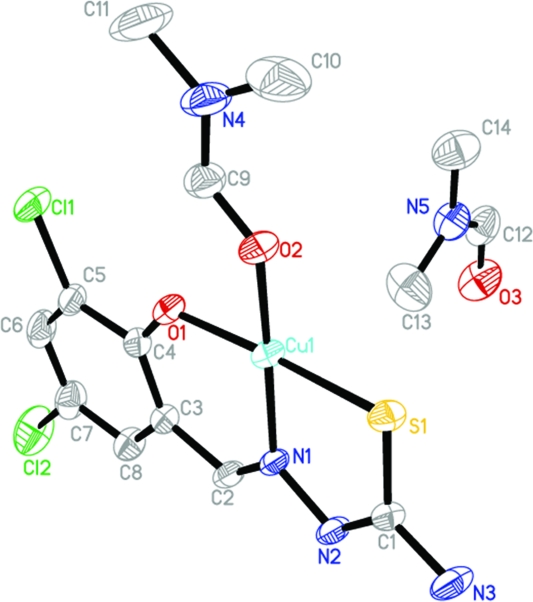
The asymmetric unit showing 30% probability displacement ellipsoids. Carbon-bound H atoms have been omitted for clarity.

**Fig. 2.**
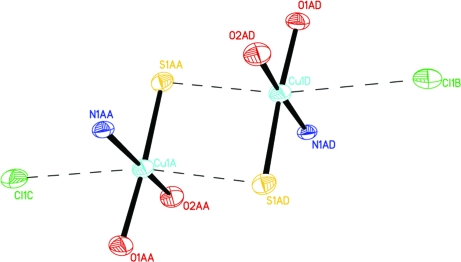
The interactions of Cl···Cu and S···Cu in the asymmetric unit one-dimensional chains.

**Fig. 3.**
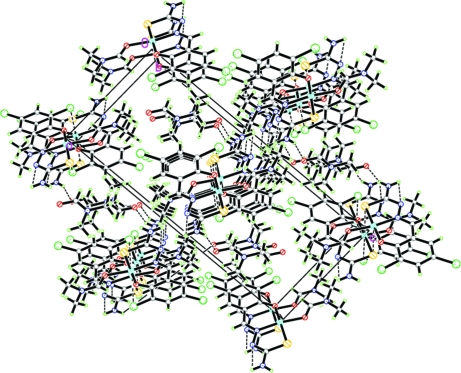
A view down the b axis, broken line showing short Cl–Cu and S···Cu contacts and hydrogen bonds.

## supplementary crystallographic information

### Comment

As a special kind of Schiff base, thiosemicarbazones and their metal complexes have become the subjects of intensive study because of their wide ranging biological activities, analytical applications and interesting chemical and structural properties (Zimmer, *et al.*, 1991). In addition, salicylaldehyde thiosemicarbazone and its substituent analogs as well as their copper complexes has been synthesized. The N—S donor also have theoretical interest, as they are capable of furnishing an environment of controlled geometry and ligand field strength.

In the title compound there is a DMF molecule coordinated through O, in addition to one N, one O and one S atom from the tridentate ligand 3,5-dichlorosalicylaldehyde thiosemicarbazone forming a slightly distorted planar square geometry (Fig. 1). In the unit cell, above and below the distorted square plane are Cl and S atoms at a long distance forming a "4 + 2" geometry. The weak interaction length of S–Cu is 2.9791 (2) Å. This bond distance are in the range of upper values for a long coordination distance (2.5–3.0 Å) in Cu(II) compounds. The length of Cl–Cu is 3.3800 (3) Å (Fig. 2). All of these facts can be seen as the result of the Jahn-Teller effect (Garcia-Orozco *et al.*, 2002). A three-dimensional network is formed through these Cl–Cu, S–Cu contacts, N–H···N and N–H···O hydrogen bonds (Fig.3).

### Experimental

An EtOH solution (15 ml) of 3,5-dichlorosalicylaldehyde (5 mmol) was added dropwise to the solution (15 ml) of thiosemicarbazide (5 mmol) and 0.75 ml acetic anhydride with stirring at *ca* 70°C for 4.5 h. The light brown precipitate was removed by filtration and recrystallized from 1:1 (*v*/*v*) MeOH/EtOH solution. Then a mixture of the ligand (0.5 mmol) and copper nitrate (0.5 mmol) in EtOH (35 ml) was stirred at *ca* 65° C for 2 h to give the desired complex. The Cu complex was dissolved in DMF, and ether slowly diffused into the DMF solution to afford almost quantitatively green crystals of the mononuclear complex at ambient temperature after several days.

### Crystal data

**Table:**

[Cu(C_8_H_5_Cl_2_N_3_OS)(C_3_H_7_NO)]·C_3_H_7_NO	*F*_000_ = 964
*M**_r_* = 471.84	*D*_x_ = 1.552 Mg m^−^^3^
Monoclinic, *P*2_1_/*n*	Mo *K*α radiation λ = 0.71073 Å
*a* = 9.4979 (10) Å	Cell parameters from 3442 reflections
*b* = 9.8057 (12) Å	θ = 2.3–26.8º
*c* = 21.744 (2) Å	µ = 1.47 mm^−^^1^
β = 94.263 (2)º	*T* = 298 (2) K
*V* = 2019.5 (4) Å^3^	Block, green
*Z* = 4	0.49 × 0.47 × 0.24 mm

### Data collection

**Table:**

Bruker SMART 1000 diffractometer	3558 independent reflections
Radiation source: fine-focus sealed tube	2587 reflections with *I* > 2σ(*I*)
Monochromator: graphite	*R*_int_ = 0.034
*T* = 298(2) K	θ_max_ = 25.0º
φ and ω scans	θ_min_ = 1.9º
Absorption correction: multi-scan(SADABS; Sheldrick, 1996)	*h* = −11→10
*T*_min_ = 0.532, *T*_max_ = 0.719	*k* = −9→11
9869 measured reflections	*l* = −23→25

### Refinement

**Table:**

Refinement on *F*^2^	Secondary atom site location: difference Fourier map
Least-squares matrix: full	Hydrogen site location: inferred from neighbouring sites
*R*[*F*^2^ > 2σ(*F*^2^)] = 0.036	H-atom parameters constrained
*wR*(*F*^2^) = 0.098	*w* = 1/[σ^2^(*F*_o_^2^) + (0.0377*P*)^2^ + 1.6178*P*] where *P* = (*F*_o_^2^ + 2*F*_c_^2^)/3
*S* = 1.06	(Δ/σ)_max_ = 0.001
3558 reflections	Δρ_max_ = 0.31 e Å^−^^3^
235 parameters	Δρ_min_ = −0.50 e Å^−^^3^
Primary atom site location: structure-invariant direct methods	Extinction correction: none

### Special details

**Table:**

Geometry. All e.s.d.'s (except the e.s.d. in the dihedral angle between two l.s. planes) are estimated using the full covariance matrix. The cell e.s.d.'s are taken into account individually in the estimation of e.s.d.'s in distances, angles and torsion angles; correlations between e.s.d.'s in cell parameters are only used when they are defined by crystal symmetry. An approximate (isotropic) treatment of cell e.s.d.'s is used for estimating e.s.d.'s involving l.s. planes.
Refinement. Refinement of *F*^2^ against ALL reflections. The weighted *R*-factor *wR* and goodness of fit *S* are based on *F*^2^, conventional *R*-factors *R* are based on *F*, with *F* set to zero for negative *F*^2^. The threshold expression of *F*^2^ > σ(*F*^2^) is used only for calculating *R*-factors(gt) *etc*. and is not relevant to the choice of reflections for refinement. *R*-factors based on *F*^2^ are statistically about twice as large as those based on *F*, and *R*- factors based on ALL data will be even larger.

### Fractional atomic coordinates and isotropic or equivalent isotropic displacement parameters (Å^2^)

**Table:**

	*x*	*y*	*z*	*U*_iso_*/*U*_eq_	
Cu1	0.54152 (4)	0.31930 (4)	0.51062 (2)	0.04237 (15)	
Cl1	0.29920 (11)	−0.05605 (11)	0.41172 (6)	0.0706 (3)	
Cl2	0.69572 (16)	−0.09500 (13)	0.24825 (6)	0.0928 (4)	
N1	0.7172 (3)	0.3290 (3)	0.46944 (12)	0.0353 (6)	
N2	0.8232 (3)	0.4197 (3)	0.48940 (14)	0.0433 (7)	
N3	0.8875 (3)	0.5886 (3)	0.55631 (15)	0.0592 (9)	
H3A	0.9666	0.5938	0.5396	0.071*	
H3B	0.8710	0.6418	0.5864	0.071*	
N4	0.1645 (3)	0.2314 (4)	0.58339 (19)	0.0736 (11)	
N5	0.3114 (4)	0.6470 (4)	0.24057 (17)	0.0673 (10)	
O1	0.4724 (2)	0.1664 (2)	0.46316 (13)	0.0515 (6)	
O2	0.3799 (2)	0.3059 (3)	0.56320 (12)	0.0536 (7)	
O3	0.4168 (4)	0.7162 (4)	0.15577 (16)	0.0890 (10)	
S1	0.63125 (9)	0.49051 (9)	0.57083 (4)	0.0431 (2)	
C1	0.7899 (3)	0.4974 (3)	0.53551 (15)	0.0386 (8)	
C2	0.7456 (3)	0.2575 (3)	0.42245 (17)	0.0427 (8)	
H2	0.8309	0.2751	0.4055	0.051*	
C3	0.6572 (3)	0.1521 (3)	0.39345 (17)	0.0430 (8)	
C4	0.5275 (4)	0.1113 (3)	0.41651 (18)	0.0440 (9)	
C5	0.4564 (4)	0.0012 (3)	0.38446 (19)	0.0496 (10)	
C6	0.5066 (5)	−0.0603 (4)	0.3345 (2)	0.0604 (11)	
H6	0.4566	−0.1317	0.3150	0.072*	
C7	0.6330 (5)	−0.0162 (4)	0.31267 (18)	0.0579 (10)	
C8	0.7069 (4)	0.0878 (4)	0.34189 (18)	0.0517 (10)	
H8	0.7916	0.1163	0.3272	0.062*	
C9	0.2784 (4)	0.2276 (4)	0.55256 (19)	0.0568 (10)	
H9	0.2831	0.1632	0.5214	0.068*	
C10	0.1465 (5)	0.3332 (6)	0.6303 (3)	0.1007 (19)	
H10A	0.2270	0.3927	0.6334	0.151*	
H10B	0.1377	0.2890	0.6692	0.151*	
H10C	0.0629	0.3854	0.6195	0.151*	
C11	0.0480 (5)	0.1379 (6)	0.5678 (3)	0.118 (2)	
H11A	−0.0330	0.1885	0.5517	0.178*	
H11B	0.0252	0.0896	0.6042	0.178*	
H11C	0.0750	0.0740	0.5374	0.178*	
C12	0.3156 (6)	0.7130 (5)	0.1874 (2)	0.0765 (14)	
H12	0.2351	0.7607	0.1731	0.092*	
C13	0.4330 (6)	0.5683 (5)	0.2633 (3)	0.1003 (18)	
H13A	0.4152	0.4732	0.2556	0.151*	
H13B	0.4508	0.5831	0.3068	0.151*	
H13C	0.5139	0.5963	0.2425	0.151*	
C14	0.1882 (5)	0.6465 (6)	0.2757 (2)	0.0926 (16)	
H14A	0.1149	0.7001	0.2547	0.139*	
H14B	0.2121	0.6847	0.3158	0.139*	
H14C	0.1557	0.5546	0.2800	0.139*	

### Atomic displacement parameters (Å^2^)

**Table:**

	*U*^11^	*U*^22^	*U*^33^	*U*^12^	*U*^13^	*U*^23^
Cu1	0.0313 (2)	0.0425 (3)	0.0537 (3)	−0.00725 (18)	0.00661 (18)	0.0006 (2)
Cl1	0.0498 (6)	0.0584 (6)	0.1019 (9)	−0.0229 (5)	−0.0066 (6)	0.0054 (6)
Cl2	0.1295 (12)	0.0781 (8)	0.0717 (8)	−0.0265 (8)	0.0131 (8)	−0.0339 (7)
N1	0.0274 (14)	0.0357 (15)	0.0427 (16)	−0.0085 (11)	0.0012 (12)	−0.0040 (13)
N2	0.0286 (14)	0.0462 (17)	0.0556 (19)	−0.0114 (12)	0.0064 (13)	−0.0158 (15)
N3	0.0365 (17)	0.072 (2)	0.071 (2)	−0.0200 (16)	0.0127 (15)	−0.0359 (18)
N4	0.039 (2)	0.089 (3)	0.094 (3)	−0.0100 (18)	0.0143 (19)	0.029 (2)
N5	0.077 (3)	0.068 (2)	0.057 (2)	−0.009 (2)	0.0072 (19)	0.0059 (19)
O1	0.0362 (13)	0.0408 (14)	0.0782 (19)	−0.0120 (11)	0.0087 (12)	−0.0043 (13)
O2	0.0386 (14)	0.0559 (16)	0.0681 (18)	−0.0111 (12)	0.0152 (12)	0.0063 (13)
O3	0.098 (3)	0.101 (3)	0.067 (2)	−0.024 (2)	−0.0003 (19)	0.0309 (19)
S1	0.0349 (5)	0.0519 (5)	0.0431 (5)	−0.0045 (4)	0.0068 (4)	−0.0034 (4)
C1	0.0312 (17)	0.044 (2)	0.0401 (19)	−0.0040 (15)	−0.0008 (15)	−0.0022 (16)
C2	0.0309 (18)	0.044 (2)	0.054 (2)	−0.0099 (15)	0.0033 (16)	−0.0054 (18)
C3	0.0384 (19)	0.0362 (19)	0.054 (2)	−0.0046 (15)	−0.0019 (16)	−0.0013 (17)
C4	0.0380 (19)	0.0337 (19)	0.059 (2)	−0.0007 (15)	−0.0046 (17)	0.0041 (17)
C5	0.044 (2)	0.037 (2)	0.066 (3)	−0.0097 (16)	−0.0119 (19)	0.0091 (19)
C6	0.070 (3)	0.039 (2)	0.068 (3)	−0.011 (2)	−0.021 (2)	−0.004 (2)
C7	0.074 (3)	0.047 (2)	0.051 (2)	−0.008 (2)	−0.007 (2)	−0.0089 (19)
C8	0.053 (2)	0.047 (2)	0.055 (2)	−0.0087 (18)	0.0024 (19)	−0.0049 (19)
C9	0.045 (2)	0.063 (3)	0.063 (3)	−0.004 (2)	0.008 (2)	0.013 (2)
C10	0.072 (3)	0.134 (5)	0.102 (4)	0.014 (3)	0.045 (3)	0.024 (4)
C11	0.048 (3)	0.141 (5)	0.167 (6)	−0.033 (3)	0.011 (3)	0.042 (5)
C12	0.089 (4)	0.066 (3)	0.072 (3)	−0.015 (3)	−0.010 (3)	0.013 (3)
C13	0.118 (5)	0.099 (4)	0.087 (4)	0.023 (3)	0.029 (3)	0.041 (3)
C14	0.083 (4)	0.119 (4)	0.077 (3)	−0.017 (3)	0.012 (3)	−0.007 (3)

### Geometric parameters (Å, °)

**Table:**

Cu1—O1	1.909 (2)	C2—H2	0.9300
Cu1—N1	1.954 (3)	C3—C8	1.398 (5)
Cu1—O2	1.985 (2)	C3—C4	1.421 (5)
Cu1—S1	2.2567 (10)	C4—C5	1.427 (5)
Cl1—C5	1.740 (4)	C5—C6	1.359 (6)
Cl2—C7	1.743 (4)	C6—C7	1.393 (6)
N1—C2	1.284 (4)	C6—H6	0.9300
N1—N2	1.388 (3)	C7—C8	1.367 (5)
N2—C1	1.316 (4)	C8—H8	0.9300
N3—C1	1.342 (4)	C9—H9	0.9300
N3—H3A	0.8600	C10—H10A	0.9600
N3—H3B	0.8600	C10—H10B	0.9600
N4—C9	1.315 (5)	C10—H10C	0.9600
N4—C10	1.446 (6)	C11—H11A	0.9600
N4—C11	1.458 (6)	C11—H11B	0.9600
N5—C12	1.328 (6)	C11—H11C	0.9600
N5—C14	1.444 (6)	C12—H12	0.9300
N5—C13	1.446 (6)	C13—H13A	0.9600
O1—C4	1.294 (4)	C13—H13B	0.9600
O2—C9	1.241 (4)	C13—H13C	0.9600
O3—C12	1.223 (6)	C14—H14A	0.9600
S1—C1	1.743 (3)	C14—H14B	0.9600
C2—C3	1.447 (4)	C14—H14C	0.9600
			
O1—Cu1—N1	93.55 (11)	C5—C6—H6	120.1
O1—Cu1—O2	90.55 (11)	C7—C6—H6	120.1
N1—Cu1—O2	172.04 (11)	C8—C7—C6	119.9 (4)
O1—Cu1—S1	176.26 (8)	C8—C7—Cl2	120.7 (3)
N1—Cu1—S1	86.04 (8)	C6—C7—Cl2	119.4 (3)
O2—Cu1—S1	89.43 (8)	C7—C8—C3	121.1 (4)
C2—N1—N2	114.1 (3)	C7—C8—H8	119.4
C2—N1—Cu1	125.1 (2)	C3—C8—H8	119.4
N2—N1—Cu1	120.8 (2)	O2—C9—N4	123.0 (4)
C1—N2—N1	113.5 (3)	O2—C9—H9	118.5
C1—N3—H3A	120.0	N4—C9—H9	118.5
C1—N3—H3B	120.0	N4—C10—H10A	109.5
H3A—N3—H3B	120.0	N4—C10—H10B	109.5
C9—N4—C10	121.6 (4)	H10A—C10—H10B	109.5
C9—N4—C11	120.2 (5)	N4—C10—H10C	109.5
C10—N4—C11	118.0 (4)	H10A—C10—H10C	109.5
C12—N5—C14	122.7 (4)	H10B—C10—H10C	109.5
C12—N5—C13	118.8 (4)	N4—C11—H11A	109.5
C14—N5—C13	118.4 (4)	N4—C11—H11B	109.5
C4—O1—Cu1	127.5 (2)	H11A—C11—H11B	109.5
C9—O2—Cu1	123.8 (3)	N4—C11—H11C	109.5
C1—S1—Cu1	94.29 (11)	H11A—C11—H11C	109.5
N2—C1—N3	116.3 (3)	H11B—C11—H11C	109.5
N2—C1—S1	125.3 (2)	O3—C12—N5	125.3 (5)
N3—C1—S1	118.4 (3)	O3—C12—H12	117.3
N1—C2—C3	126.0 (3)	N5—C12—H12	117.3
N1—C2—H2	117.0	N5—C13—H13A	109.5
C3—C2—H2	117.0	N5—C13—H13B	109.5
C8—C3—C4	120.6 (3)	H13A—C13—H13B	109.5
C8—C3—C2	116.9 (3)	N5—C13—H13C	109.5
C4—C3—C2	122.5 (3)	H13A—C13—H13C	109.5
O1—C4—C3	124.8 (3)	H13B—C13—H13C	109.5
O1—C4—C5	119.6 (3)	N5—C14—H14A	109.5
C3—C4—C5	115.6 (3)	N5—C14—H14B	109.5
C6—C5—C4	123.0 (4)	H14A—C14—H14B	109.5
C6—C5—Cl1	119.3 (3)	N5—C14—H14C	109.5
C4—C5—Cl1	117.7 (3)	H14A—C14—H14C	109.5
C5—C6—C7	119.8 (3)	H14B—C14—H14C	109.5
			
O1—Cu1—N1—C2	7.1 (3)	N1—C2—C3—C4	−2.9 (6)
O2—Cu1—N1—C2	127.9 (7)	Cu1—O1—C4—C3	3.3 (5)
S1—Cu1—N1—C2	−176.6 (3)	Cu1—O1—C4—C5	−176.6 (2)
O1—Cu1—N1—N2	−174.1 (2)	C8—C3—C4—O1	−178.8 (3)
O2—Cu1—N1—N2	−53.3 (9)	C2—C3—C4—O1	3.1 (5)
S1—Cu1—N1—N2	2.2 (2)	C8—C3—C4—C5	1.2 (5)
C2—N1—N2—C1	176.6 (3)	C2—C3—C4—C5	−176.9 (3)
Cu1—N1—N2—C1	−2.3 (4)	O1—C4—C5—C6	179.0 (3)
N1—Cu1—O1—C4	−7.1 (3)	C3—C4—C5—C6	−1.0 (5)
O2—Cu1—O1—C4	179.7 (3)	O1—C4—C5—Cl1	−1.6 (4)
S1—Cu1—O1—C4	−90.6 (13)	C3—C4—C5—Cl1	178.5 (3)
O1—Cu1—O2—C9	−7.4 (3)	C4—C5—C6—C7	0.1 (6)
N1—Cu1—O2—C9	−128.4 (7)	Cl1—C5—C6—C7	−179.4 (3)
S1—Cu1—O2—C9	176.3 (3)	C5—C6—C7—C8	0.7 (6)
O1—Cu1—S1—C1	82.5 (12)	C5—C6—C7—Cl2	−179.5 (3)
N1—Cu1—S1—C1	−1.24 (14)	C6—C7—C8—C3	−0.5 (6)
O2—Cu1—S1—C1	172.22 (14)	Cl2—C7—C8—C3	179.7 (3)
N1—N2—C1—N3	−179.1 (3)	C4—C3—C8—C7	−0.5 (6)
N1—N2—C1—S1	0.9 (4)	C2—C3—C8—C7	177.7 (3)
Cu1—S1—C1—N2	0.6 (3)	Cu1—O2—C9—N4	−171.5 (3)
Cu1—S1—C1—N3	−179.4 (3)	C10—N4—C9—O2	3.2 (7)
N2—N1—C2—C3	177.6 (3)	C11—N4—C9—O2	179.0 (4)
Cu1—N1—C2—C3	−3.5 (5)	C14—N5—C12—O3	−179.7 (5)
N1—C2—C3—C8	178.9 (3)	C13—N5—C12—O3	−1.9 (8)

### Hydrogen-bond geometry (Å, °)

**Table:**

*D*—H···*A*	*D*—H	H···*A*	*D*···*A*	*D*—H···*A*
N3—H3A···N2^i^	0.86	2.14	2.992 (4)	170
N3—H3B···O3^ii^	0.86	2.08	2.886 (4)	157

Symmetry codes: (i) −*x*+2, −*y*+1, −*z*+1; (ii) *x*+1/2, −*y*+3/2, *z*+1/2.
